# The Impact of Laboratory Automation on the Time to Urine Microbiological Results: A Five-Year Retrospective Study

**DOI:** 10.3390/diagnostics14131392

**Published:** 2024-06-29

**Authors:** Antonios Kritikos, Guy Prod’hom, Damien Jacot, Antony Croxatto, Gilbert Greub

**Affiliations:** 1 Institute of Microbiology, Lausanne University Hospital (CHUV), University of Lausanne, 1005 Lausanne, Switzerland; guy.prodhom@chuv.ch (G.P.); damien.jacot@chuv.ch (D.J.); antony.croxatto@ne.ch (A.C.); 2 Unité d’Infectiologie, Département de Médecine, Hôpital de Fribourg HFR, 1752 Villars-sur-Glâne, Switzerland; 3 ADMED Microbiology, 2000 La Chaux-de-Fonds, Switzerland

**Keywords:** total automation, laboratory automation, turnaround time, time to results, urine cultures

## Abstract

Total laboratory automation (TLA) is a valuable component of microbiology laboratories and a growing number of publications suggest the potential impact of automation in terms of analysis standardization, streaking quality, and the turnaround time (TAT). The aim of this project was to perform a detailed investigation of the impact of TLA on the workflow of commonly treated specimens such as urine. This is a retrospective observational study comparing two time periods (pre TLA versus post TLA) for urine specimen culture processing. A total of 35,864 urine specimens were plated during the pre-TLA period and 47,283 were plated during the post-TLA period. The median time from streaking to identification decreased from 22.3 h pre TLA to 21.4 h post TLA (*p* < 0.001), and the median time from streaking to final validation of the report decreased from 24.3 h pre TLA to 23 h post TLA (*p* < 0.001). Further analysis revealed that the observed differences in TAT were mainly driven by the contaminated and positive samples. Our findings demonstrate that TLA has the potential to decrease turnaround times of samples in a laboratory. Nevertheless, changes in laboratory workflow (such as extended opening hours for plate reading and antibiotic susceptibility testing or decreased incubation times) might further maximize the efficiency of TLA and optimize TATs.

## 1. Introduction

Over recent years, clinical microbiology laboratories have been facing an increase in overall testing volumes and growing challenges resulting from the detection of multidrug-resistant microorganisms, increased numbers of immunocompromised patients, and infection control demands due to new microbes or reemerging pathogens [1,2,3]. A constant need is therefore expressed for improved quality, increased productivity, and faster diagnostics with reduced turnaround times (TATs) [4]. In this context, laboratory automation appears an appealing option given that the bulk of the workload in culture-based analysis relies on manual processes such as specimen handling, media plate inoculation, culture reading, and antibiotic susceptibility testing, which all can be automatized [3,5].

Although it has been 30 years since the first automation systems were introduced, technological advances have only recently allowed a wider implementation of automated systems worldwide [6,7,8]. Since then, a growing number of publications have demonstrated the benefits in terms of improved standardization (enhanced quality and higher reproducibility of bacterial cultures), improved laboratory efficiency, increased workplace safety, and reduced long-term costs [9,10,11,12,13,14]. While the positive impact of total laboratory automation (TLA) on the laboratory workflow has been shown in numerous studies, the opposite results have been observed as well. A few recent studies have associated automation with a slightly increased TAT from receipt until the final identification of positive samples and/or an increased total processing time [15,16]. However, a detailed evaluation of TLA covering all aspects of analytical workflow is lacking. Such an approach could facilitate targeting the analytical steps for workflow improvement and TLA optimization.

Urine specimens are one of the most common specimens processed in microbiology laboratories (being the second most common in our laboratory after blood cultures). Manual processing (inoculation, transfer of plates between benchtops and incubators, etc.) occupy approximately 30% of a technologist’s time [4,5], and therefore there is great potential for improvement thanks to automated systems. Our laboratory obtained a fully automated system (BD Kiestra TLA^TM^, Sparks, MD, USA) in 2017. After the implementation of TLA, we set up a procedure for sample processing based on digitally reading solid media cultures after 18 h of incubation (for urine samples), followed by immediate identification and antibiotic susceptibility testing (AST) when deemed necessary. Consequently, urine samples were able to be almost exclusively processed through our TLA system, thus allowing a representative evaluation of the different analytical steps until the generation of the final microbiological report. The aim of this project was to perform a detailed investigation of the impact of TLA on the workflow of commonly treated specimens such as urines, and to identify possible bottlenecks that would allow further reductions in the time to results.

## 2. Materials and Methods

### 2.1. Setting

This study took place in Lausanne University Hospital (CHUV), a one-thousand-bed tertiary hospital in Lausanne, Switzerland. The technical staff of our laboratory processed urine samples daily from 8:00 to 17:00. Urines were not processed during the weekend besides the inoculation of new samples and AST read-outs on Saturdays.

### 2.2. Study Design

This is a retrospective observational study comparing two periods in which two different approaches were used for urine specimen culture processing (pre TLA versus post TLA). During the pre-TLA period (from January 2016 to October 2017), urine specimens were processed manually for the incubation and reading of plates (specimens were plated with the WASP^TM^ Copan system), while during the post-TLA period (from June 2018 to December 2020), inoculation, incubation, and digital reading of the specimens were processed automatically with the BD Kiestra^TM^ (Franklin Lakes, NJ, USA) TLA system. From our analysis, we excluded the 2-month period spanning the implementation of the TLA system in our laboratory (November and December 2017). We moreover accounted for a period of adaptation for our technical staff to the TLA system during the first semester of 2018 (January to May 2018), during which regular technical controls and updates were held.

The urine specimens’ culture and identification were the same throughout the study. Urine specimens (10 µL) were inoculated on chromogenic agar (BD^TM^ CHROMagar^TM^ Orientation, Franklin Lakes, NJ, USA) and/or selective and differential medium (BD^TM^ MacConkey II agar, Franklin Lakes, NJ, USA) when deemed necessary. Plates were held overnight (ranging from 14 h to 24 h) at 35 °C in CO_2_ in a conventional laboratory incubator during the pre-TLA period and in a Kiestra^TM^ incubator post TLA. It is worth noting that in the pre-TLA period, samples that were plated last the day before were the first to be treated the day after, and thus the incubation time varied between 14 h and 24 h. During the post-TLA period, all plates were digitally imaged with the Kiestra^TM^ system at 18 h. Identification was performed using MALDI-TOF MS (Bruker BioTyper^®^, Billerica, MA, USA), except for *E. coli*, where a typical colony appearance in the chromogenic plates (pink dry colonies) was considered sufficient according to laboratory’s standard operating procedures.

To evaluate the impact of TLA on TATs, laboratory data for all urine cultures were extracted from our laboratory information system (MOLIS CGM Group©, CompuGroup Medical, Nanterre, France). The dates and times of all analytical steps were captured. Cultures were subsequently stratified in three categories. Sterile cultures included any culture with a categorical interpretation that was “sterile” or “negative”. Contaminated cultures included those with a categorical interpretation of “contamination”, “contaminating flora”, or “normal flora”. Finally, positive cultures included any culture not considered “contaminated” and presenting bacterial growth irrespectively of the colony count threshold. Not all positive cultures were considered clinically significant based on current clinical standards, but a diagnostic workup was nevertheless performed. Based on their categorical interpretation, some culture results were subjected to a preliminary report, to AST, and/or to a final review and validation by a laboratory supervisor. Of note, our urine specimen management algorithm was modified in spring 2018 (the beginning of our post-TLA period evaluation). That change was meant to simplify the management of contaminated samples (urine specimens with multiple species warranted no further evaluation from this moment) and therefore accelerate the identification process, since less subcultures and identifications were performed thereafter. All of the changes made to our laboratory organization and workflow during the study course are shown in Figure S1.

**Figure 1:**
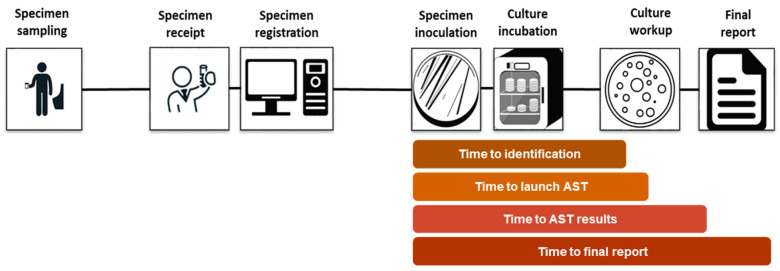
Urine culture workflow and turnaround times captured during the study period.

Turnaround times were calculated using the date and time of the specimen’s inoculation in the microbiology laboratory as a starting point for the analysis. Endpoints for the TATs were defined as the date and time recorded at the completion of each specified milestone of the urine culture workflow. The following TAT milestones were recorded: time until bacterial identification; time until the launch of AST and/or time to AST results (for positive cultures); and, ultimately, time to final report validation (Figure 1).

The following exclusion criteria were applied to all TAT results: analyses performed as part of a quality control procedure or samples belonging to clinical studies; cultures with positive results that yielded any *Candida* spp.; samples with missing recorded timepoints and TAT outliers. Outliers were defined as reports with a TAT 4 standard deviations above the median or a TAT of less than 6 h. The excluded outliers were specimens that did not follow standard processing and identification pathways. Finally, samples belonging to the TLA implementation period or the immediate post-implementation period were not analyzed.

### 2.3. Statistical Analyses

Standard descriptive statistics were used to summarize the data, such as the mean, standard deviation (SD), median, interquartile range (IQR), and percentage. The time to each milestone was analyzed and reported as hours in decimal format. All data manipulation and analysis procedures were conducted using R v.4.1.3 (www.cran.r-project.org, accessed on 5 February 2024) and RStudio-2022.02.1–461. Statistical comparisons were undertaken using the Kruskal–Wallis or Wilcoxon non-parametric rank sum test for quantitative variables and Pearson’s Chi-squared test for qualitative variables where appropriate.

### 2.4. Ethics Statement

This project was a quality assurance project of the microbiology laboratory and did not require approval by the local ethics committee. Data included in the database were de-identified before access and no personal information was stored in the study database.

## 3. Results

### 3.1. Analysis Cohort and Workflow

**Figure 2:**
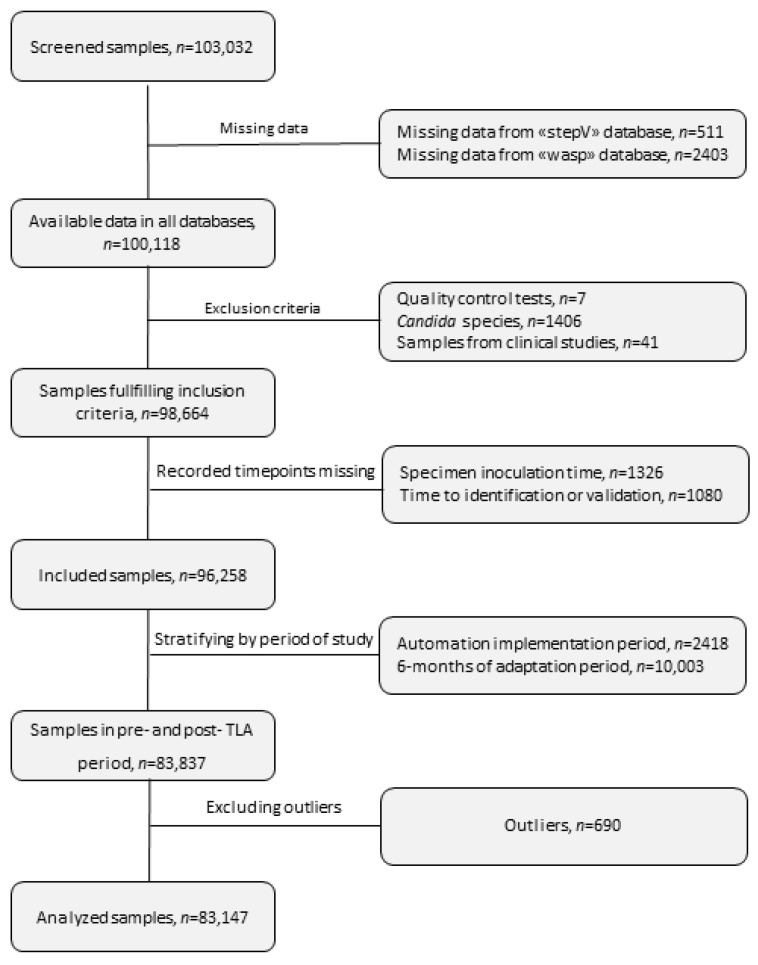
Flowchart of included samples. Three databases were investigated for this project, namely “Infoculture”, “Wasp”, and “StepV”. The Infoculture database provided the samples for screening, as well as information on epidemiological and microbiological data. The Wasp database contained information regarding the samples’ inoculation times, and the StepV database contained information on consecutive steps of the workflow until the final validation of the result.

**Figure 3:**
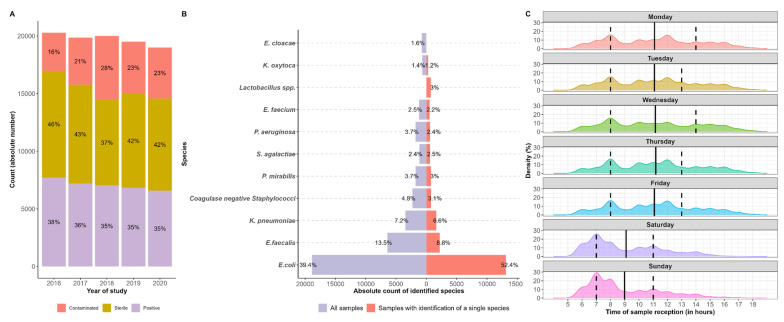
Demographics of specimens included in the study. (**A**) Results of urine specimens included in the study. (**B**) Distribution of microorganisms isolated from patient urine cultures. (**C**) Density plots showing the daily distribution of urine samples (workload) throughout the week. (Solid black lines represent mean values and dashed lines represent the interquartile range (IQR).)

A total of 98,664 urine samples fulfilled the inclusion criteria. Figure 2 summarizes the inclusion/exclusion criteria and the sample filtration process. Table 1 describes the samples and the patients’ characteristics. Overall, approximately 36% (*n* = 35,351) of all samples turned out positive, among which 71% were monomicrobial infections and 29% were polymicrobial infections (Table 1). Contaminated and sterile cultures represented 22% and 42%, respectively, of all 98,664 urines investigated. A slight decrease in positive-sample rates was observed over time, with a concomitant increase in contaminated samples (Figure 3A). Eleven species accounted for >80% of all positive urine cultures (Figure 3B). As expected, *E. coli*, *E. faecalis*, and *K. pneumoniae* were most commonly isolated from the positive samples, followed by coagulase-negative *Staphylococci*, other Gram-negative *Enterobacteriales*, and non-fermenting bacteria (Figure 3B). In order to assess the workload of our workflow during the day, we recorded the specimens’ reception time for every day of the week (Figure 3C). We note a two-peak distribution of the reception of samples during the weekdays (early in the morning and at noon) and a one-peak distribution during the weekend (early in the morning) (Figure 3C).

### 3.2. Process Timing Metrics

**Figure 4:**
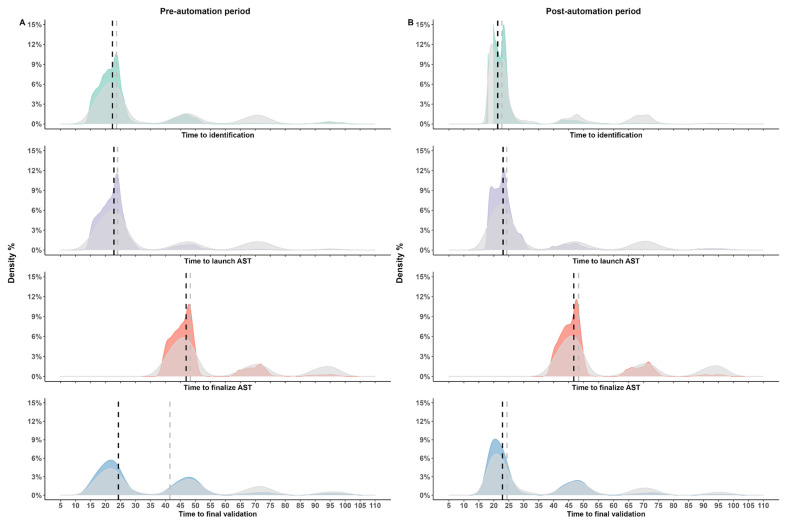
Density plots of TAT distribution during the two study periods. Panel (**A**) shows the TAT distribution on the pre-automation period and panel (**B**) on the post-automation period. The colored density plots show TATs from weekday samples, while the superimposed grey-colored plots show all samples combined (weekend included). The black and grey-colored dashed lines show the median TATs from the weekday samples vs. all samples included, respectively.

A total of 35,864 urine specimens were plated with the Copan WASP during the pre-TLA period (circa 1600 samples/month) and 47,283 urines were plated with the BD InoqulA instrument (component of BD Kiestra^TM^ TLA system) (circa 1525 samples/month). To evaluate the impact of TLA on urine samples’ TATs, we first examined the impact of the weekend on the time to results. Figure 4 shows the distribution of the different TATs stratified by study period. It is noteworthy that in both the pre- and post-TLA periods, the median TATs were longer when all samples were taken into account, compared to when only samples treated on weekdays were considered (TATs until final validation: 41.9 h vs. 24.3 h pre TLA and 24.5 h vs. 23 h post TLA, respectively). The weekend had a major effect on the TAT until final validation of a sample’s result (69.7 h in the pre-TLA period and 66.8 h in the post-TLA period, respectively. Indeed, due to our laboratory’s organization, urine samples are not prioritized during the weekend in cases where the laboratory has a heavy workload. In fact, only the inoculation of new urine samples is performed during the weekend, as well as AST reading on Saturdays. We were concerned that this internal laboratory organization might affect the comparison of TATs, and therefore samples received during the weekend were not included in subsequent analyses.

**Figure 5:**
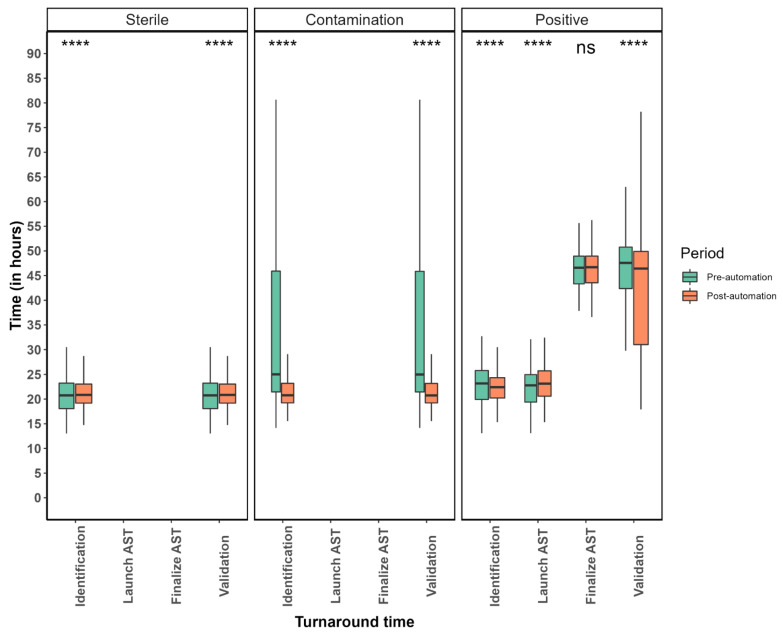
Boxplots of TATs stratified by the specimen result and study period. ns: non-significant, **** = *p* < 0.0001.

**Figure 6:**
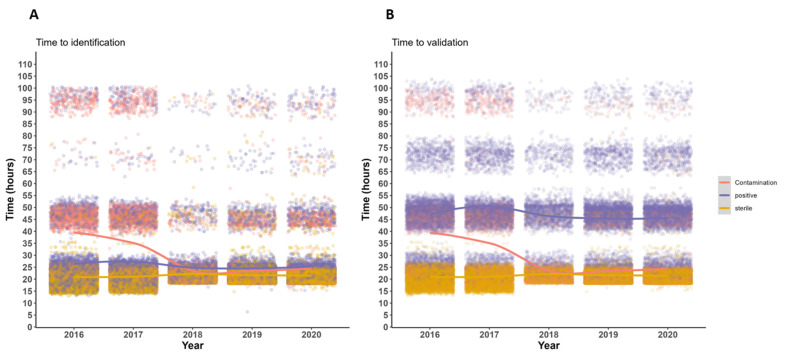
Scatterplot of TATs until identification and until final validation throughout the study period. Panel (**A**) shows the time until identification and panel (**B**) the time until final validation. Each dot represents one observation (one analyzed sample). The smooth colored lines show the general trends over time.

The median time from streaking to identification decreased from 22.3 h pre TLA to 21.4 h post TLA (*p* < 0.001) (Table 2). Similarly, the median time from streaking to final validation of the report decreased from 24.3 h pre TLA to 23 h post TLA (*p* < 0.001) (Table 2). While in the post-TLA period, the time from streaking to launching AST was longer (from 22.8 h pre TLA to 23.2 h post TLA) (*p* < 0.001), we did not observe any statistically significant differences in the time needed to finalize AST (from 46.9 h pre TLA to 47 h post TLA) (*p* = 0.065) (Table 2). Further analysis revealed that the observed differences in TAT were mainly driven by the contaminated and positive samples (Figure 5, Figure 6 and Figure S2). Besides the reduced TATs during the post-TLA period, we moreover observed decreased interquartile ranges for the different TATs (except for the time taken until the final validation of positive samples), suggesting improved standardization with the use of TLA (Figure 5). When we examined positive samples stratified by the number of identified microorganisms (monomicrobial vs. polymicrobial samples), we observed that the polymicrobial samples were those that resulted in increased time to launch AST and had a higher dispersion of TATs (Figure S3). Finally, we intended to identify potential bottlenecks of our workflow that could potentially be improved to further reduce TATs. Figure S4 compares the median TATs until pathogen identification or until the final validation of the report based on the samples’ reception times. Specific days or day timeslots were therefore identified that were associated with a longer time to results. Thus, samples received on either Fridays or Saturdays or samples received very early or very late during weekdays were associated with a longer TAT until validation.

## 4. Discussion

Early etiological diagnosis and appropriate therapy are critical in achieving successful outcomes for patients [17,18]. From this perspective, global efforts are concentrating on the development of faster AST methods, allowing for the rapid identification of the disease-causing pathogen and its antibiotic susceptibility [19]. Nevertheless, optimization of the current methods and workflow could also lead to faster diagnostics, and in that sense, laboratory automation represents an appealing option [3]. Laboratory automation in clinical microbiology laboratories has gained much interest during the last decade, with a growing amount of research evaluating its potential impact [3,20].

In this study, we evaluated the impact of BD Kiestra TLA^TM^ on the workflow of our laboratory. We compared two time periods (pre and post TLA implementation) that included more than 80,000 urine specimens. Urine specimens are one of the most common specimen types submitted to clinical microbiology laboratories (the second most common in our laboratory) [10]. Our study compared a semi-automated process with a fully automated one, providing evidence that transitioning from semi-automation to full automation is beneficial. A thorough analysis of all analytical steps from identification to final report validation was performed. Previously published data have suggested that the implementation of TLA can quantifiably improve many aspects of culture-based urine analysis and/or reduce TATs [5,9,10,11,21]. In line with such data, our study demonstrates a reduction in TAT for the most important aspects of the workflow besides the time taken to launch or finalize AST [9,10,11,21]. Yarbough et al. and Strauss et al. have already demonstrated in previous studies that TLA resulted in increased time from receipt to inoculation regarding urine samples and a slightly higher TAT for the identification of positive samples [15,16]. Authors from those studies argued that the workflow for these specimens included a batching step in which specimens were on a tabletop rack waiting to be loaded rather than in the hands of personnel during active hands-on-time. In our study, we report an increased TAT related to the AST of positive samples resulting from polymicrobial specimens. Polymicrobial specimens need subcultures of their different bacterial morphologies to be carried out before AST is performed, and therefore this slows down the process. Moreover, in our case, a similar batching step occurred with the specimens with polymicrobial results since the technical staff needed to recall the plates on the working bench in order to evaluate them manually (and this is usually carried out by batch in our laboratory). An increased number of plates recalled on the working bench can result in a traffic jam of plates on the TLA system’s chain and therefore increase the waiting time before the technical staff handle the samples.

In most of the previous studies, reductions in the TAT were explained by sterile and/or positive samples [9,10,11,15,16]. In our study, we also observed a slight reduction in the TAT for positive samples, but not to a degree that is foreseen to have significant benefits on patient care. We moreover observed a statistically significant reduction in the TAT for contaminated samples, but this is more likely explained by the urine management algorithm change, which happened concomitantly, rather than being an effect of the automation itself. This algorithm change during the study period could eventually also explain the slight progressive decrease in the number of positive samples against the slight increase in the number of contaminated samples.

Interestingly, multiple previous studies have demonstrated improved standardization of urine specimen handling after TLA [13,14,15,22]. In our study, we also observed overall shorter interquartile ranges of the different TATs and reduced TAT dispersion. In our laboratory, plates are automatically photographed via our TLA system at predetermined intervals (at 18 and 24 h of incubation) and these photographs are reviewed at the earliest opportunity on the day shift. In our particular setting (not operating as a 24 h/7 laboratory), this standardization allows for sufficient culture growth from inoculation to the first plate reading.

However, our study exhibits a few limitations. First, our post-TLA study period fell during the emergence of the COVID-19 pandemic crisis. While we do not anticipate any significant impact of the pandemic on our bacteriology laboratory, this might explain the slight decrease in the total number of received specimens throughout the post-TLA years. Secondly, but most importantly, the retrospective nature of this study imposed us to apply multiple filtering levels to our data and therefore we excluded an important proportion of samples (17%). In this same context, we highlight some changes in our laboratory organization and workflow during the course of the study which might have influenced the results. The change in the algorithm for contaminated urine specimens, for example, most likely influenced the TATs for this specific group of samples. In the pre-TLA period, plates were incubated from 14 h up to 24 h before their evaluation. In the post-TLA period, the incubation time was standardized at 18 h before evaluation. This difference might explain the small effect of TLA implementation observed in our laboratory in comparison to previous studies that report a more significant reduction in TATs. Lastly, due also to the retrospective nature of our data, we were not able to evaluate the pre-analytical aspects of our workflow and we had to use the inoculation as the starting point of our analyses.

In conclusion, the results of the present study show that processing urine specimens with TLA can slightly reduce turnaround times and improve the laboratory workflow. Nevertheless, TLA implementation itself cannot automatically eliminate human bottlenecks and may necessitate changes in the workflow in order to fully exploit TLA’s potential. The feasibility of re-organizing the staffing or extending the laboratory’s opening hours should be evaluated within the current system’s constraints. Digital processes for the imaging and time-reading of plates could also be adapted based on the presence of laboratory technicians in the laboratory. Besides existing laboratory automation modules, a TLA system also needs to integrate microbial identification using MALDI-TOF and antibiotic susceptibility testing [23,24]. Becton Dickinson have developed a prototype for automated MALDI-TOF identification coupled to antibiotic susceptibility testing [23,25], but currently only the identification module is available. Copan have also developed a fully automated antimicrobial disk diffusion susceptibility testing method with automated interpretation using an expert system (Radian^TM^) [26,27]. Finally, the integration of all automated systems with the laboratory information system would allow a linear flow of all the information related to a specimen’s processing. In that sense, the implementation of artificial intelligence algorithms for automated sample validation within the TLA system would further significantly decrease technicians’ hands-on-time and TATs [14,28,29,30]. It is also important to note that an observed reduction in a specimen’s TAT does not necessarily translate to an improved outcome for the patient or even a change in the patient’s care. Further prospective studies are needed to evaluate the impact of laboratory automation on patient care. However, this will only make sense when the overall processing time is further reduced by additional layers of automation, including (i) automated sample release; (ii) automated colony picking, coupled with automated preparation of the inoculum used for AST; and (iii) automated release of the expected antibiotic susceptibility profiles (corresponding to wild-type strains of the corresponding species).

## Figures and Tables

**Table 1:** Characteristics of the samples of included patients.

	2016 (*n* = 20,290)	2017 (*n* = 19,860)	2018 (*n* = 20,006)	2019 (*n* = 19,510)	2020 (*n* = 18,998)	Overall (*n* = 98,664)	*p*-Value
Age (years)	52 (±23)	52 (±23)	52 (±23)	53 (±23)	53 (±24)	52 (±23)	<0.001
Gender (number of females)	12,409 (61.2%)	11,937 (60.1%)	12,008 (60.0%)	11,446 (58.7%)	11,176 (58.8%)	58,976 (59.8%)	<0.001
Category of result							<0.001
Contaminated samples	3313 (16.3%)	4086 (20.6%)	5545 (27.7%)	4521 (23.2%)	4420 (23.3%)	21,885 (22.2%)	
Sterile cultures	9256 (45.6%)	8584 (43.2%)	7427 (37.1%)	8162 (41.8%)	7999 (42.1%)	41,428 (42.0%)	
Positive cultures	7721 (38.1%)	7190 (36.2%)	7034 (35.2%)	6827 (35.0%)	6579 (34.6%)	35,351 (35.8%)	
Number of identified species for positive cultures							<0.001
1	5632 (73%)	5208 (72%)	4998 (71%)	4756 (70%)	4440 (67%)	25,034 (71%)	
2	1624 (21%)	1522 (21%)	1541 (22%)	1537 (23%)	1541 (23%)	7765 (22%)	
3	384 (5%)	393 (5.5%)	404 (5.7%)	453 (6.6%)	511 (7.8%)	2145 (6%)	
≥4	81 (1%)	67 (0.9%)	91 (1.3%)	81 (1.2%)	87 (1.3%)	407 (1%)	

Median (SD) values are shown for quantitative variables and absolute numbers (%) are shown for qualitative variables. Kruskal–Wallis rank sum test was performed for quantitative variables and Pearson’s Chi-squared test was performed for qualitative variables.

**Table 2:** Turnaround times stratified per group and study period.

Turnaround Time	Group											
	Sterile			Contaminated			Positive			Overall		
	Pre TLA (*n* = 16,129)	Post TLA (*n* = 19,275)	*p*-Value	Pre TLA (*n* = 6505)	Post TLA (*n* = 11,413)	*p*-Value	Pre TLA (*n* = 13,230)	Post TLA (*n* = 16,595)	*p*-Value	Pre TLA (*n* = 35,864)	Post TLA (*n* = 47,283)	*p*-Value
From streaking to identification	20.8 [5.16]	20.9 [3.86]	<0.001	38.8 [25.4]	20.8 [3.99]	<0.001	23.3 [6.01]	22.5 [4.17]	<0.001	22.3 [5.69]	21.4 [4.01]	<0.001
From streaking to the start of AST	NA	NA	NA	NA	NA	NA	22.8 [5.67]	23.2 [5.29]	<0.001	22.8 [5.67]	23.2 [5.29]	<0.001
From streaking to finalizing AST	NA	NA	NA	NA	NA	NA	46.9 [6.00]	47.0 [6.10]	0.065	46.9 [6.00]	47.0 [6.10]	0.065
From streaking to final validation	20.8 [5.16]	20.9 [3.86]	<0.001	30.7 [25.4]	20.8 [3.98]	<0.001	48.0 [9.00]	46.9 [9.90]	<0.001	24.3 [26.7]	23.0 [23.8]	<0.001

NA: “Not applicable”.

## Data Availability

The data of this project are not publicly available but can be made available upon reasonable request to the authors.

## Supplementary Materials

The following supporting information can be downloaded at: https://www.mdpi.com/article/10.3390/diagnostics14131392/s1, Figure S1: Timeline of laboratory and workflow changes during the study period.; Figure S2: Scatterplot of TAT to launch and finalize AST throughout the study period.; Figure S3: Boxplots of positive urine specimens TAT, stratified by period of study and category of positive samples.; Figure S4: Heatmap showing TAT until identification and final validation of the specimen’s report stratified by weekday and time of specimen’s inoculation.
