# Supplementary figures and images for: The Impact of Laboratory Automation on the Time to Urine Microbiological Results: A Five-Year Retrospective Study
Source: Diagnostics (Basel). 2024 Jun 29;14(13):1392. doi: 10.3390/diagnostics14131392 (PMC11240889; doi:10.3390/diagnostics14131392)

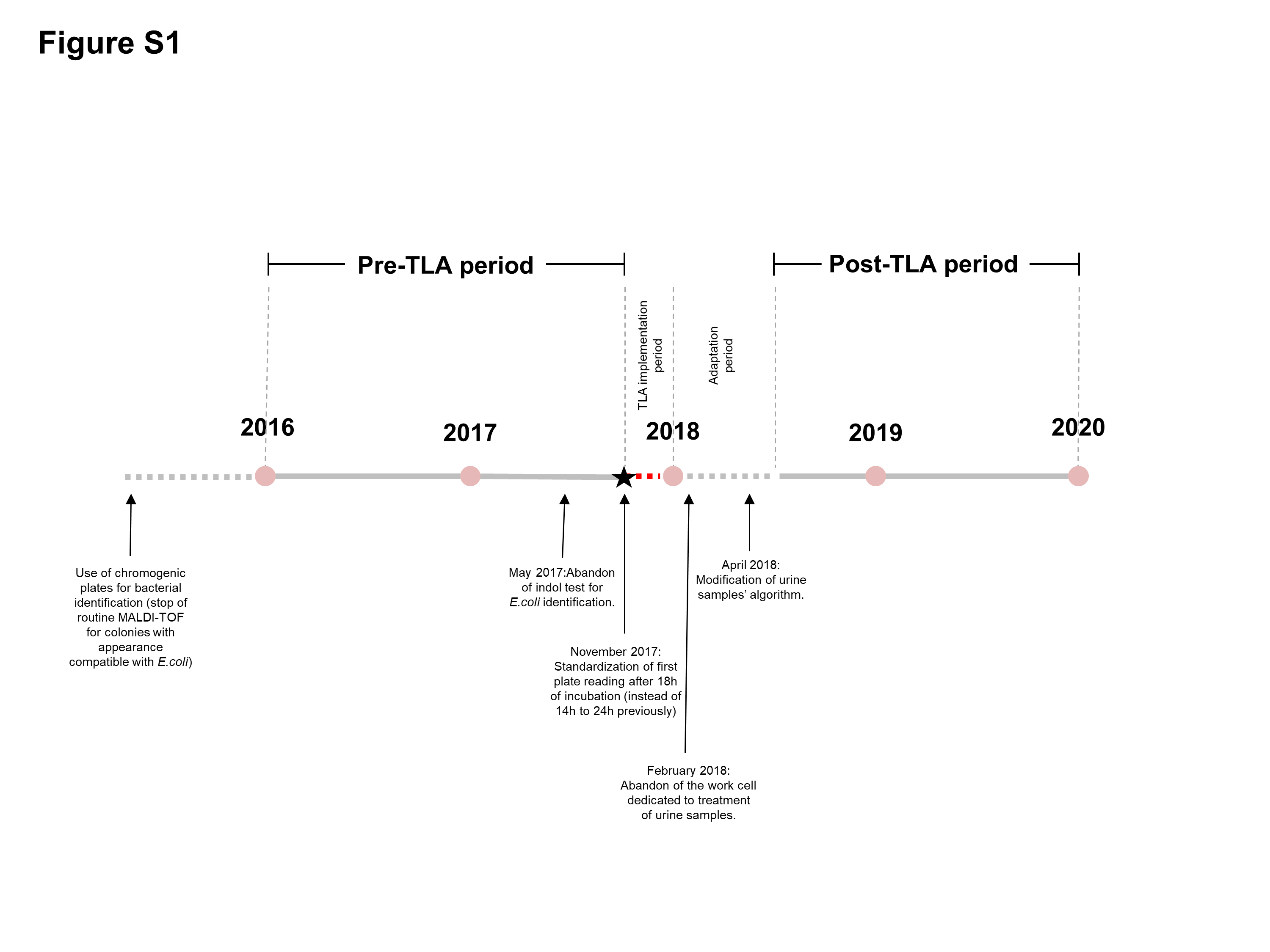

Supplement: Supplementary file 1 [file diagnostics-14-01392-s001.zip › FigureS1.TIF]

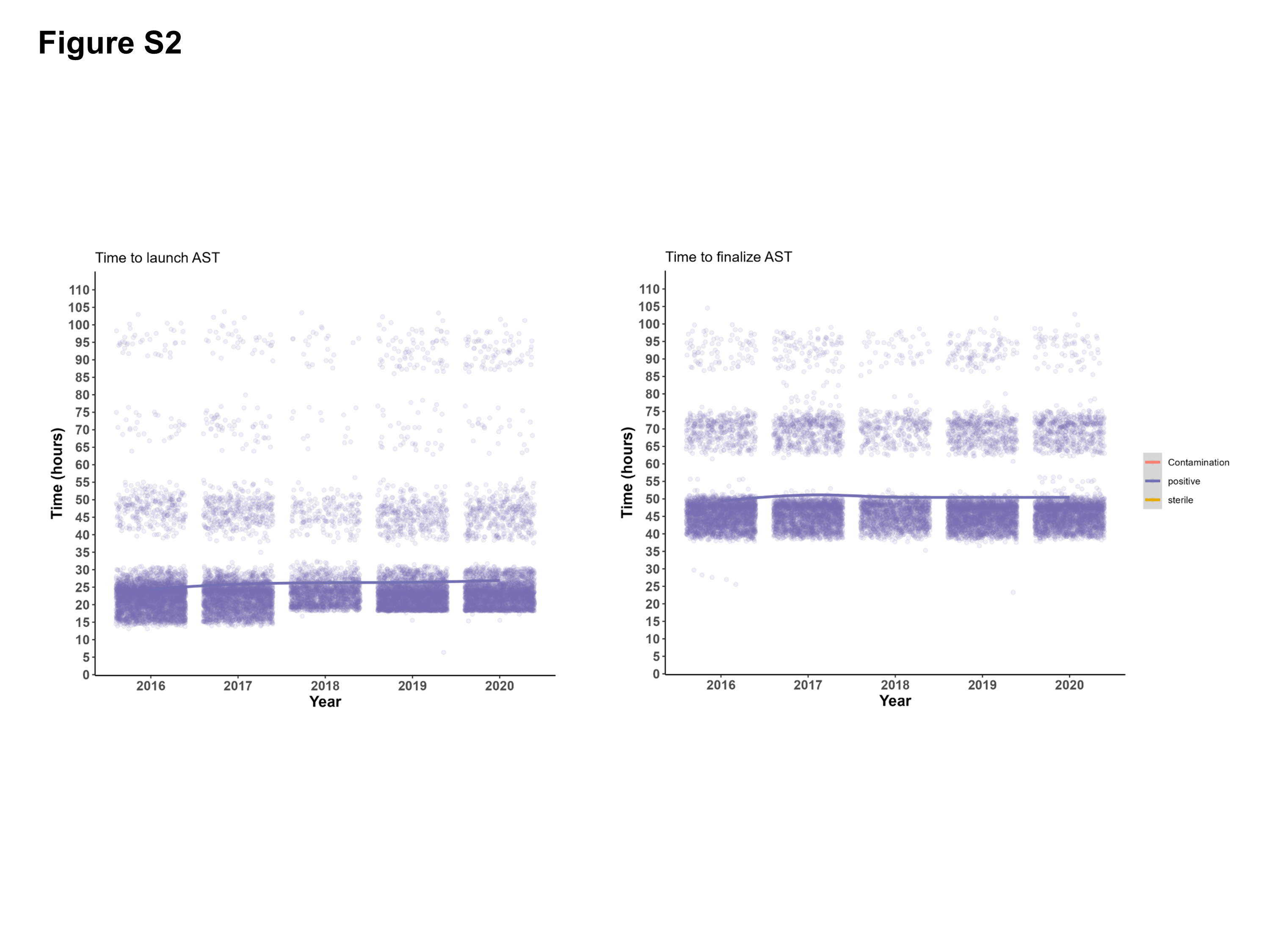

Supplement: Supplementary file 1 [file diagnostics-14-01392-s001.zip › FigureS2.TIF]

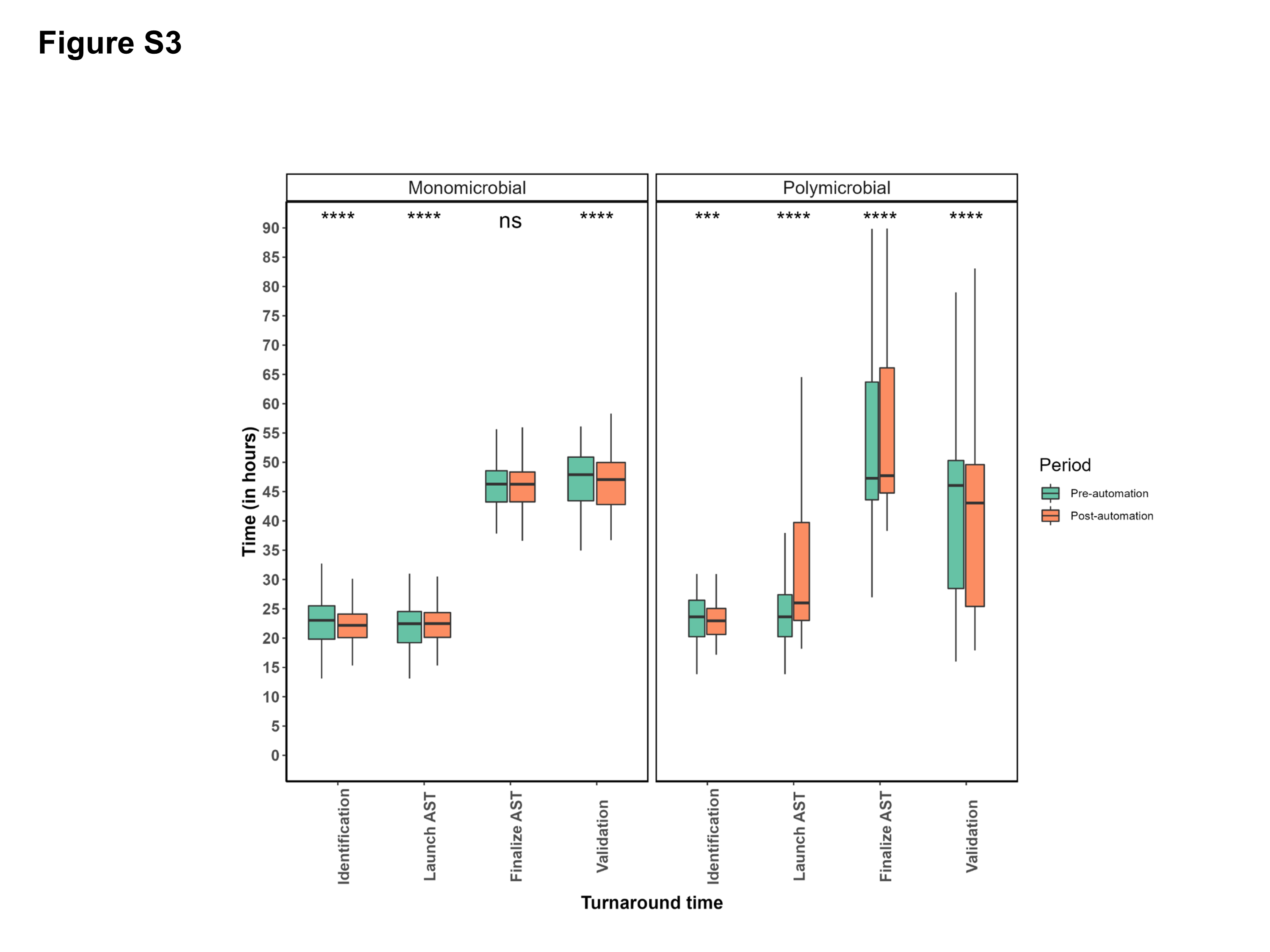

Supplement: Supplementary file 1 [file diagnostics-14-01392-s001.zip › FigureS3.TIF]

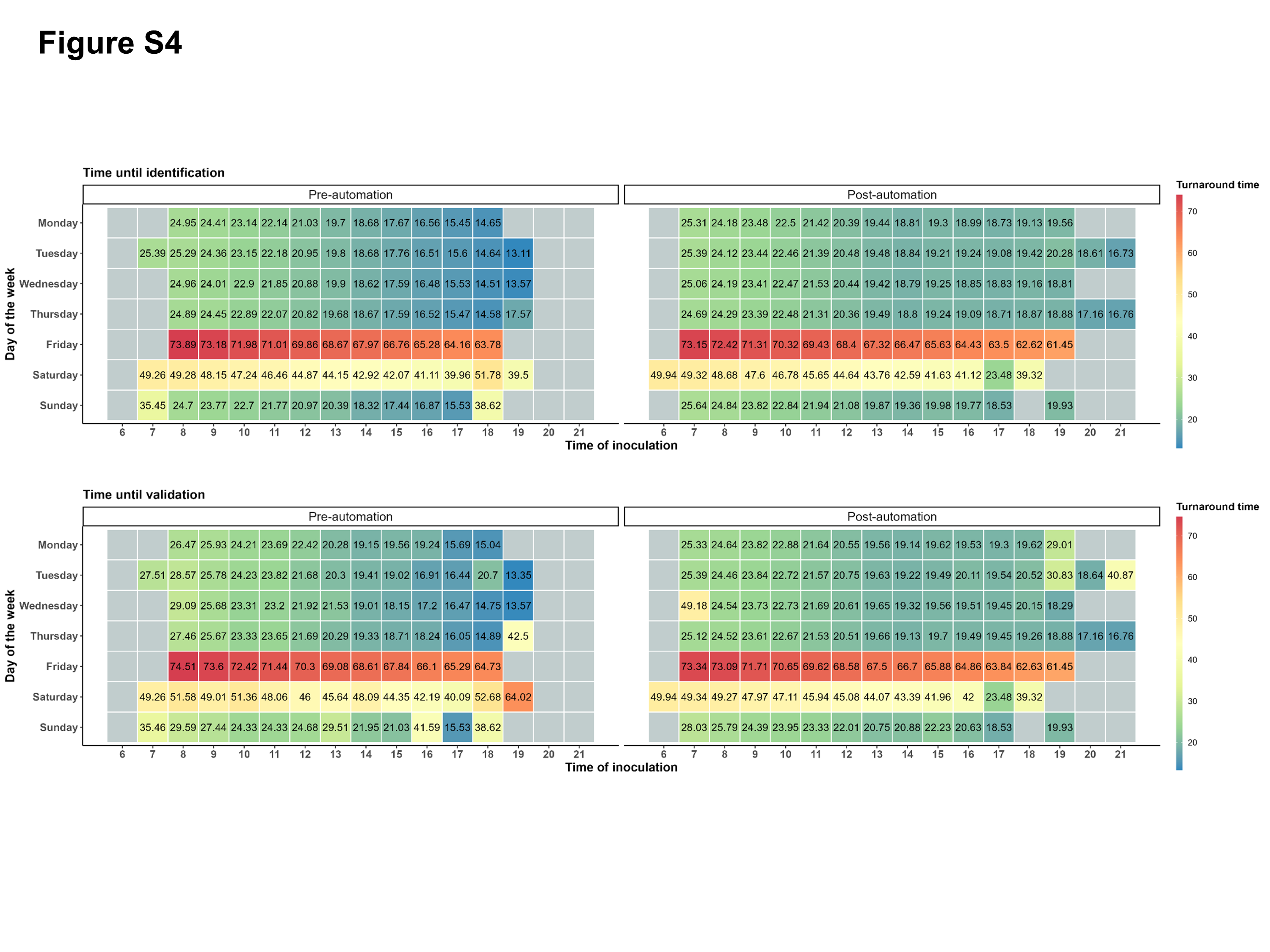

Supplement: Supplementary file 1 [file diagnostics-14-01392-s001.zip › FigureS4.TIF]
